# Inositol for the prevention of neural tube defects: a pilot randomised controlled trial

**DOI:** 10.1017/S0007114515005322

**Published:** 2016-02-05

**Authors:** Nicholas D. E. Greene, Kit-Yi Leung, Victoria Gay, Katie Burren, Kevin Mills, Lyn S. Chitty, Andrew J. Copp

**Affiliations:** 1 Newlife Birth Defects Research Centre, Institute of Child Health, University College London, London WC1N 1EH, UK; 2 Genetics, Genomics and Metabolism Programme, Institute of Child Health, University College London, London WC1N 1EH, UK

**Keywords:** Neural tube defects, Pregnancy supplements, Inositol, Folic acid supplementation

## Abstract

Although peri-conceptional folic acid (FA) supplementation can prevent a proportion of neural tube defects (NTD), there is increasing evidence that many NTD are FA non-responsive. The vitamin-like molecule inositol may offer a novel approach to preventing FA-non-responsive NTD. Inositol prevented NTD in a genetic mouse model, and was well tolerated by women in a small study of NTD recurrence. In the present study, we report the Prevention of Neural Tube Defects by Inositol (PONTI) pilot study designed to gain further experience of inositol usage in human pregnancy as a preliminary trial to a future large-scale controlled trial to evaluate efficacy of inositol in NTD prevention. Study subjects were UK women with a previous NTD pregnancy who planned to become pregnant again. Of 117 women who made contact, ninety-nine proved eligible and forty-seven agreed to be randomised (double-blind) to peri-conceptional supplementation with inositol plus FA or placebo plus FA. In total, thirty-three randomised pregnancies produced one NTD recurrence in the placebo plus FA group (*n* 19) and no recurrences in the inositol plus FA group (*n* 14). Of fifty-two women who declined randomisation, the peri-conceptional supplementation regimen and outcomes of twenty-two further pregnancies were documented. Two NTD recurred, both in women who took only FA in their next pregnancy. No adverse pregnancy events were associated with inositol supplementation. The findings of the PONTI pilot study encourage a large-scale controlled trial of inositol for NTD prevention, but indicate the need for a careful study design in view of the unwillingness of many high-risk women to be randomised.

Neural tube defects (NTD) are severe congenital malformations, affecting 0·5–2/1000 pregnancies^(^ ^1^ ^)^. A proportion of NTD cases can be prevented by peri-conceptional folic acid (FA) supplementation, as demonstrated in the 1991 Medical Research Council (MRC) Vitamin Study^(^ ^2^ ^)^. However, NTD recurred in 1 % of pregnancies supplemented with 4 mg FA in this trial, suggesting that some NTD cases are non-responsive to FA. Although food fortification programmes have generally resulted in a reduction in NTD prevalence, compared with historical pre-fortification frequencies, a proportion of NTD persists^(^ ^3^ ^)^. In part, this may be due to a group of women who have erythrocyte folate concentrations that are ‘suboptimal’ for NTD prevention. Indeed, even in a FA-fortified environment, women who do not consume FA-containing supplements are more likely to have low blood folate concentrations than those who take such supplements^(^ ^4^ ^)^. On the other hand, women who do consume dietary FA supplements, in a fortified population, can nevertheless go on to have NTD-affected pregnancies^(^ ^5^ ^,^ ^6^ ^)^, suggesting that increased FA dosage does not always provide additional protection against NTD. Novel therapies are needed to improve NTD prevention, by encompassing FA-non-responsive cases that currently cannot be prevented.

Inositol deficiency is the only ‘vitamin’ deficiency that leads to NTD in rodent embryos^(^ ^7^ ^)^; even FA deficiency does not cause NTD in mice in the absence of genetic predisposition^(^ ^8^ ^)^. Inositol supplementation during pregnancy reduced the frequency of NTD in the FA-non-responsive mouse NTD model *curly tail* (*Grhl3* gene)^(^ ^9^ ^,^ ^10^ ^)^, a finding that has been replicated in another laboratory^(^ ^11^ ^)^. A mechanism of action of inositol was identified, in which diacylglycerol is generated by the action of phospholipase C on phosphatidylinositol, activating protein kinase C isoforms *β*1 and *γ* ^(^ ^12^ ^)^. This normalises neural tube closure by correcting a *Grhl3*-dependent cell proliferation defect in the ventral tissues underlying the closing neural tube^(^ ^13^ ^,^ ^14^ ^)^.

In humans, peri-conceptional supplementation with inositol and FA was studied in a cohort of twelve Caucasian mothers each of whom had at least one previous NTD-affected pregnancy, despite almost all having taken FA supplements^(^ ^15^ ^)^. This apparently ‘FA-non-responsive’ cohort then underwent fifteen further pregnancies while taking inositol plus FA, resulting in the birth of seventeen babies without NTD. Typically quoted NTD recurrence risks are 3·1 % for women following a single affected pregnancy and 11·8 % after two previous affected pregnancies^(^ ^16^ ^)^. The lack of NTD among these seventeen births, together with the finding of significantly lower inositol concentrations in the blood of mothers carrying NTD fetuses than in normal pregnancies^(^ ^17^ ^)^, supports the idea that inositol could play a key role in the prevention of NTD.

Despite this previous work, there has been no controlled trial of pregnancy supplementation in humans to determine whether inositol might provide additional protection against NTD, beyond that achieved by FA alone. The MRC Vitamin Study recruited approximately 600 pregnancies (all in women with a previous NTD pregnancy) into each of its two combined trial arms: FA supplementation *v*. no FA supplementation; this required a multi-national recruitment effort^(^ ^2^ ^)^. Anticipating a similar or larger sample size requirement for a controlled trial of inositol supplements, we chose to begin by carrying out a small pilot study in the UK, in order to gain further experience of using inositol in human pregnancy and to assess the feasibility of performing a double-blind randomised controlled trial.

In the present study, we describe the findings of the Prevention of Neural Tube Defects by Inositol (PONTI) pilot study. This study was designed as a pilot evaluation of inositol, in combination with FA, in preparation for a later, fully powered controlled clinical trial to provide definitive evidence on the efficacy of inositol. The PONTI study recruited women with a history of NTD pregnancy with the following objectives: (i) to explore the prospects for randomisation of women at high risk of NTD to inositol+FA supplementation *v*. placebo+FA; (ii) to follow-up the largest cohort to date of high-risk women using inositol supplements during a subsequent pregnancy; (iii) to focus on adverse maternal and pregnancy outcomes, in order to determine whether inositol usage is safe in pregnancy; and (iv) to trial a newly developed, MS-based assay^(^ ^18^ ^)^ for measuring inositol concentration in urine and blood samples, as a potential means of evaluating compliance with supplementation.

## Methods

This study with human subjects was conducted according to the guidelines laid down in the Declaration of Helsinki, and all the procedures involving human subjects were approved by the National Research Ethics Service Committee London – Bloomsbury (REC Reference: 06/Q0508/87). Written informed consent was obtained from all subjects. Clinical trial registration was with Clinicaltrials.gov, with the following registration identification number: NCT00452829 (https://clinicaltrials.gov/ct2/show/NCT00452829).

### Participants

Women were eligible to join the study if they had a history of previous NTD-affected pregnancy and were planning to become pregnant again. Exclusion criteria were as follows: (i) women who were unable to provide informed consent or did not have a general practitioner/obstetrician in the UK; (ii) maternal age outside of 18–40 years; (iii) cases where the previous affected child had abnormalities in addition to NTD, suggestive of a syndromic association with specific aetiology; and (iv) women who were epileptic and/or taking anti-epileptic medications. Participants who did not become pregnant within 12 months of starting supplementation were withdrawn from the study.

Recruitment was achieved by requesting referral from obstetricians, fetal medicine specialists and clinical geneticists across the UK, through media publicity to encourage self-referral and through the advocacy of charities including SHINE (Spina Bifida, Hydrocephalus, Information, Networking, Equality; http://www.shinecharity.org.uk/) and ARC (Antenatal Results and Choices; http://www.arc-uk.org/). Women who contacted the study centre, and appeared eligible based on initial enquiry, were asked whether they would consent to participation in a randomised double-blind trial structure. Those who indicated that they were likely to consent to randomisation were invited to undergo a structured telephone interview and to complete a questionnaire. These women were subsequently sent a study information pack, consent form and two urinary collection tubes. Once the signed consent form and pre-supplementation urine samples were returned by post, the women received the first batch of inositol/placebo and FA tablets. The second urine sample was requested to be collected 6 weeks after starting supplementation.

Eligible women who declined randomisation did not undergo the detailed telephone interview and did not complete the questionnaire. They were nevertheless followed-up by telephone and email, with the aim of determining the details of their chosen peri-conceptional supplementation regimen and the outcome of their next pregnancy.

A group of ten, young-adult research staff volunteers, both men and non-pregnant women, were recruited with informed consent to participate in a week-long study of urinary and blood inositol concentrations, before, during and after oral inositol supplementation (1·3 g/d).

### Study duration

Recruitment of women subjects commenced on 8 September 2009 and ended in early 2013, once more than 100 women (the original target number) had contacted the study centre. Completion date for the study was 27 September 2013, when all women who had been randomised and were pregnant had delivered their babies. The randomisation code was broken in December 2013. Pregnancies reported by non-randomised women were included in the analysis only if they occurred during the period between September 2009 and September 2013.

### Interventions

In the randomisation study, all women were prescribed 5 mg FA daily: the standard UK supplement for pregnancies at high risk of NTD. Women randomised to the inositol group additionally received 1 g inositol (two 500 mg tablets), whereas those in the control group received 1 g placebo (two tablets) instead of inositol. Inositol and placebo tablets were manufactured in the Pharmacy of University College London Hospitals National Health Service (NHS) Foundation Trust. The active ingredient was myo-inositol obtained from Tsuno Rice Fine Chemicals. Excipients were maize starch, povidone and magnesium stearate. Placebo tablets had the same formulation except that inositol was replaced by dextrose monohydrate (Tate & Lyle). FA tablets were obtained from Alpha-Pharma Limited (Actavis UK). The daily consumption of supplements occurred unsupervised in the participants’ homes, according to instructions supplied with the tablets. Supplementation was initiated before conception and continued until the end of the 12th post-menstrual week. Non-randomised women procured their own peri-conceptional supplements, but all of them asked for and received details from the study team regarding the supplementation protocol that was used in the randomisation study. We obtained as accurately as possible details of the supplementation regimens that the non-randomised women had followed.

### Outcomes

The primary outcome measures were as follows: (i) to determine the feasibility of recruiting, randomising and following-up women with a history of NTD pregnancy, who were embarking upon a further pregnancy; (ii) to gather preliminary data on NTD recurrence frequency in the UK trial setting; (iii) to determine the safety of inositol administration during the peri-conceptional period and first trimester of pregnancy; and (iv) to trial a newly developed, MS-based assay^(^ ^18^ ^)^ for measuring inositol concentration in urine and blood samples, as a potential means of evaluating compliance with supplementation. Pregnancy outcomes (live birth *v*. miscarriage; normal *v*. NTD) were determined from direct reports by the randomised women subjects, with corroboration from their supervising doctors. Adverse maternal and pregnancy outcomes were determined from a structured reporting sheet supplied to the women subjects at registration. In the non-randomised group, pregnancy outcomes were determined from direct reports by the women subjects. No attempt was made to formally collect data on adverse events from the non-randomised women.

### Randomisation and blinding

Randomisation and blinding were performed in the Pharmacy of the Great Ormond Street Hospital (GOSH) for Children NHS Foundation Trust. The randomisation code was provided by the Clinical Trials Unit at the University of Nottingham (CTU Trial Ref 0709). Investigators who were in contact with the randomised women subjects, as well as laboratory analysts and pregnancy outcome assessors, were blind to the treatment group. The protocol allowed for access to the randomisation code by GOSH Pharmacy and one investigator in the event of unexpected adverse reactions. This facility was not required, and the randomisation code was broken only at the end of the study.

### Inositol assay and statistical analysis

Inositol concentrations in urine and plasma samples were determined by MS as described previously^(^ ^18^ ^)^. Values were not normally distributed, and non-parametric statistical analysis was performed, using the Mann–Whitney *U* test, computed using SigmaStat version 3.5.

## Results

A goal of the PONTI study was to gauge to what extent a future randomised controlled trial of inositol supplementation for NTD prevention would face recruitment challenges. We considered two different scenarios: first, women might be unwilling to take inositol during pregnancy in line with the prevalent view after the thalidomide experience that ‘medications’ should be avoided during the early stages of pregnancy; second, women at high risk of NTD might be unwilling to be randomised to a placebo (non-inositol) group, if they believed that inositol could help them have a baby unaffected by NTD (given that FA appeared to have ‘failed’ to protect their previous pregnancy). To investigate these two possible scenarios, we attempted to randomise as many women as possible within the study.

### Feasibility of recruitment to a randomised, controlled trial of inositol supplementation

**Fig. 1:**
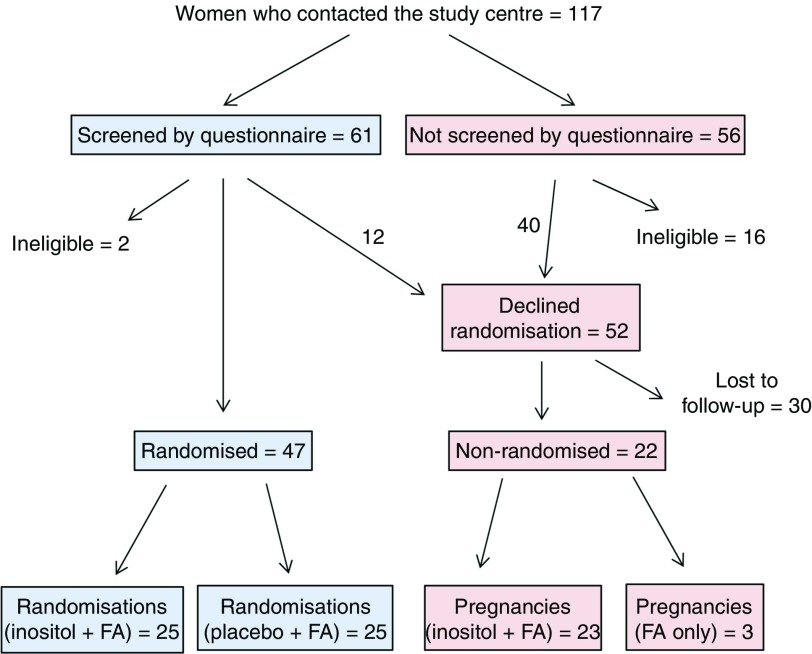
Flow of women subjects through the Prevention of Neural Tube Defects by Inositol study. Number of subjects are shown at each stage of the study. For outcome of randomisations and pregnancies, see Table 1. FA, folic acid.

Fig. 1 shows the flow of women through the study. Of 117 women who made contact with the study centre, sixty-one underwent structured questionnaire screening by telephone. Of these women, fifty-nine proved eligible on detailed screening, whereas two women were ineligible. Of the eligible women, forty-seven chose to undergo randomisation, and were initially randomised to either the inositol+FA group (twenty-two subjects) or the placebo+FA group (twenty-five subjects). Therefore, we were able to randomise 40 % of those who originally made contact with the study centre, and 77 % of those who underwent detailed questionnaire screening. As the study progressed, one woman in the placebo+FA group had a healthy baby and wished to become pregnant again. She was re-randomised to the inositol+FA group, making a total number of twenty-three subjects in this group. Moreover, two women in the inositol+FA group had miscarriages, and they were re-randomised for subsequent pregnancies to the inositol+FA group (i.e. same group as first pregnancy), making a total number of twenty-five randomisations in this group. Therefore, the final analysis was based on a total of fifty randomisations: twenty-five to the inositol+FA group and twenty-five to the placebo+FA group.

In total, fifty-two of the 117 women who contacted the study centre declined randomisation, despite satisfying the eligibility criteria on initial enquiry or questionnaire screening (Fig. 1). Many of these women indicated that they did not wish to be randomised to the placebo+FA group and expressed the intention of using inositol supplements in their next pregnancy. In all, twenty-two of these women subsequently informed the study team that they had become pregnant during the study period, and they provided details of their supplementation regimen and subsequently of their pregnancy outcome. Of these non-randomised women, nineteen took peri-conceptional supplementation with inositol+FA, whereas three women took peri-conceptional supplementation with FA alone.

We conclude that fewer than half of the eligible women who contacted the study team were prepared to undergo randomisation with regard to inositol supplementation in their next pregnancy. However, many of the women who declined randomisation subsequently self-supplemented with inositol+FA. This indicates that women at high-risk of NTD are generally prepared to take an ‘untested’ supplement if they believe it will increase their likelihood of having a baby unaffected by NTD. In fact, so strong was this desire that many declined randomisation in order to avoid the 50 % chance of being prescribed a placebo.

### Previous pregnancy histories

The data are summarised in Table 1. Of the forty-seven women who were randomised, forty-six had previously experienced a single pregnancy complicated by spina bifida (thirty-one cases) or anencephaly (fifteen cases), whereas one woman reported two previous cases of anencephaly. The breakdown of previous NTD pregnancy history was closely similar between the randomised groups: fourteen spina bifida and eight anencephaly in the inositol+FA group and seventeen spina bifida and eight anencephaly (including the woman with two anencephalics) in the placebo+FA group. Interestingly, the twenty-two women who declined randomisation, and whose next pregnancy outcomes could be determined, had overall experienced more previous NTD pregnancies: six women (all of them took inositol in their next pregnancy) had two or more previous NTD, whereas sixteen women had single previous NTD.

**Table 1:** Pregnancy outcomes in the Prevention of Neural Tube Defects by Inositol pilot study

	Randomised		Non-randomised	
	Inositol+FA	Placebo+FA	Inositol+FA*	FA alone*
No. of subjects	22 (23†)	25	19	3
Previous NTD pregnancies	SB×1=14 Anenc×1=8	SB×1=17 Anenc×1=7 Anenc×2=1	SB×1=8 Anenc×1=3 Enceph×1=1 NTD×1=1 NTD×2−3=6	Anenc×1=2 NTD×1=1
No. of randomisations	25‡	25	N/A	N/A
No. of pregnancies	18	21	23§	3
No. of established pregnancies	14	19	21	3
Without NTD	14	18	21||	1
With NTD	0	1¶	0	2¶
No. of other pregnancies	4	2	2	0
Miscarriage	3	2	2	0
Ectopic	1	0	0	0
No. of women who failed to become pregnant	6	3	N/A**	N/A
No. of lost to follow-up or withdrew from study	1	1	N/A	N/A
Adverse maternal events reported	Interruption of menstrual cycle (one case); bad morning sickness and gestational diabetes (one case)	Bad morning sickness (one case)	N/A	N/A
Adverse pregnancy outcomes reported	Caesarean section (four cases, including two non-elective)	Caesarean section (six cases, all elective); birth at <36 weeks (one case)	N/A	N/A

FA, folic acid; NTD, neural tube defects; SB, spina bifida; ×1 etc., number of previous NTD pregnancies experienced by the subjects; Anenc: anencephaly; Enceph: encephalocele.

* Dosages of inositol and FA were determined by direct reports from the women subjects.

† Twenty-two subjects were initially randomised, with one additional subject re-randomised to this group after birth of a baby on the placebo+FA group.

‡ Two women were re-randomised to this group for a second pregnancy following miscarriages.

§ Four women reported two successive pregnancies while on supplements during the study period.

|| Includes one baby with hypoplastic right heart malformation.

¶ Anenc diagnosed by ultrasound and pregnancy terminated.

** N/A: not applicable to non-randomised subjects.

### Pregnancy outcomes

The data are summarised in Table 1. Of the twenty-five randomisation events in the inositol+FA group, fourteen led to the birth of normal babies (eight girls, six boys), three ended in miscarriage and one ended in ectopic pregnancy. Of the remaining seven randomised subjects, six failed to conceive within a year, and one woman was lost to follow-up. There were no NTD among the fourteen established pregnancies in the inositol+FA group.

Of the twenty-five randomisation events in the placebo+FA group, eighteen normal babies were born (thirteen girls, five boys), whereas one subject underwent termination of pregnancy following an ultrasound diagnosis of anencephaly. This subject had a history of one previous pregnancy with spina bifida. Two miscarriages occurred and three of the remaining four subjects failed to conceive within a year, whereas one subject withdrew from the study. Therefore, there was one NTD among nineteen established pregnancies in the placebo+FA group.

Of the non-randomised group, nineteen women reported having twenty-three pregnancies (four women had two pregnancies each) following peri-conceptional supplementation with inositol+FA during the study period. Reported inositol dose varied from 0·5 to 1·35 g, with most women taking 1 g daily (as in the randomised group); the usual FA dose was 5 mg, although one woman took 15 mg FA daily. Of these twenty-three pregnancies, twenty produced normal babies, two ended in miscarriage and one baby was born with a serious congenital heart defect (hypoplastic right heart). Three pregnancies were reported by women who declined randomisation and took only 5 mg FA in the subsequent pregnancy. Of these, one resulted in a normal baby, whereas two pregnancies were terminated because of anencephaly. Both of these women had experienced a single previous pregnancy with anencephaly.

In summary, three cases of NTD (all anencephaly) occurred among the fifty-seven established pregnancies that were reported by women who satisfied the eligibility criteria for this study and were either randomised (one NTD) or non-randomised (two NTD).

### Supplementation periods before conception and neurulation

Erythrocyte folate concentrations rise gradually following the onset of daily supplementation and can take several weeks to reach an ‘optimum’ value (e.g. 1000 nmol/l)^(^ ^19^ ^)^. Among our randomised women subjects, the mean number of days that trial supplements were taken before conception were 105·4 (SEM 19·6) (inositol+FA group) and 74·5 (SEM 19·9) (placebo+FA group), a statistically non-significant difference (*t* test; *P*=0·28). The randomised subject whose fetus had anencephaly took supplements (placebo+FA) for 74 d before conception. We conclude that women received trial supplements that included ‘high dose’ FA on average for 6 weeks or more before conception, and thus for 9 weeks or more before neurulation, which begins 3 weeks after conception. Moreover, the majority of subjects reported that they were already taking FA-containing supplements before entering the study. It seems likely, therefore, that the erythrocyte folate concentrations in the majority of randomised women in this study had reached an ‘optimum’ level by the 3rd week after conception. Our findings with blood inositol assay (see below) suggest a more rapid response to supplementation than for FA, suggesting that subjects had achieved enhanced inositol concentrations well in advance of the onset of neurulation.

### Safety of peri-conceptional supplementation with inositol

No unexpected events were reported by the randomised study subjects. Pregnancies that ended in miscarriage and caesarean section were the most common events encountered (Table 1), whereas there was one ectopic pregnancy in the inositol+FA group and one NTD in the placebo+FA group. Six women failed to become pregnant in the inositol+FA group and four in the placebo+FA group (*P*=0·47). Adverse event data were not collected systematically from the non-randomised women. In conclusion, there was no evidence of any adverse outcomes specifically associated with peri-conceptional inositol supplementation.

### Inositol assay using urine and blood samples

**Fig. 2:**
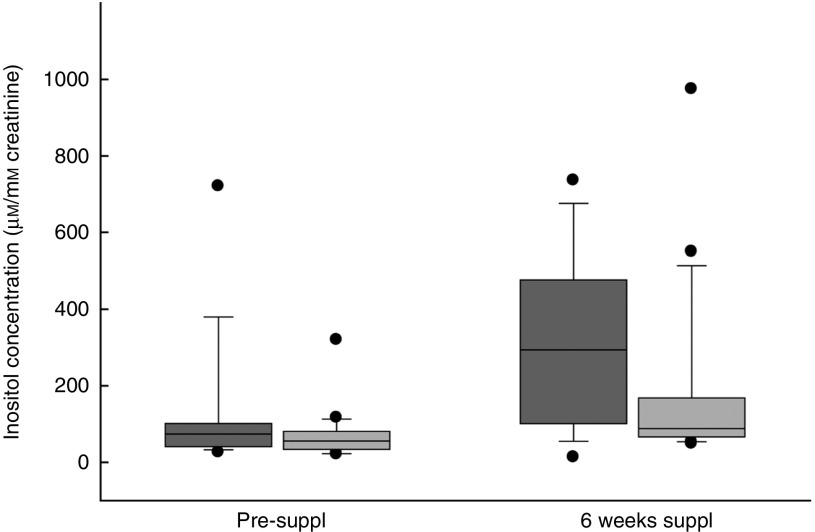
Inositol concentration in the urine of the Prevention of Neural Tube Defects by Inositol pilot study subjects. Inositol was quantified by MS^(^ ^18^ ^)^ in urine samples collected before the beginning of supplementation (pre-suppl) or 6 weeks after the subjects had received their first batch of supplements (6 weeks suppl). Box and whisker plots define the median flanked by (bottom to top) the 10th, 25th, 75th and 90th percentile values. Outlying values are represented by dots. Sample numbers: study group, eleven (pre-suppl), eleven (6 weeks); control group, seventeen (pre-suppl), sixteen (6 weeks). For statistical analysis, see Table 2. 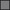, Study group (inositol+folic acid (FA)); 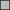, control group (placebo+FA).

Inositol concentrations for the randomised women subjects, before pregnancy, were determined by MS^(^ ^18^ ^)^, using urine samples collected at home and posted to the study centre. Inositol is stable in urine for at least 72 h at ambient temperature and for longer if stored at −20°C^(^ ^18^ ^)^. Large variations in urinary inositol concentration were, nevertheless, noted between women subjects (Fig. 2). Non-parametric statistical analysis (Table 2) revealed a significant increase in median inositol concentration from pre-supplementation to 6 weeks’ supplementation in both the inositol+FA group (73·9–294·1 µm/mm creatinine) and the placebo+FA group (55·8–88·4 µm). This could indicate that one or more women in the placebo+FA group in fact took inositol outside the study protocol. However, the median inositol concentration at 6 weeks was more than 3-fold greater in the inositol+FA group (294·1 µm) than in the placebo+FA group (88·4 µm). Moreover, the individual increase in inositol concentration from pre-supplementation to 6 weeks’ supplementation was more than 6-fold greater in the inositol+FA group (256·8 µm) than in the placebo+FA group (41·0 µm). Hence, the statistically significant increase from pre-supplementation to 6 weeks in the placebo+FA group could have resulted from individual differences in urinary inositol excretion, rather than non-compliance with the study protocol.

**Table 2:** Statistical analysis of urinary inositol concentrations (µm/mm creatinine) (Medians and interquartile ranges (IQR))

	Inositol+FA		Placebo+FA		
	Median	IQR	Median	IQR	*P* *
Pre-suppl	73·9	40·9–101·9	55·8	34·0–81·1	0·347
6 weeks suppl	294·1	101·5–477·0	88·4	66·3–168·4	0·089
*P* *	0·036†		0·012†		
6 weeks minus pre-suppl‡	256·8	25·3–364·8	41·0	4·9–110·2	0·246

Suppl, supplementation.

* From Mann–Whitney *U* tests.

† Significant at *P*<0·05.

‡ Median change (IQR) in inositol concentration from pre-suppl to 6 weeks, calculated for each individual women subject.

**Fig. 3:**
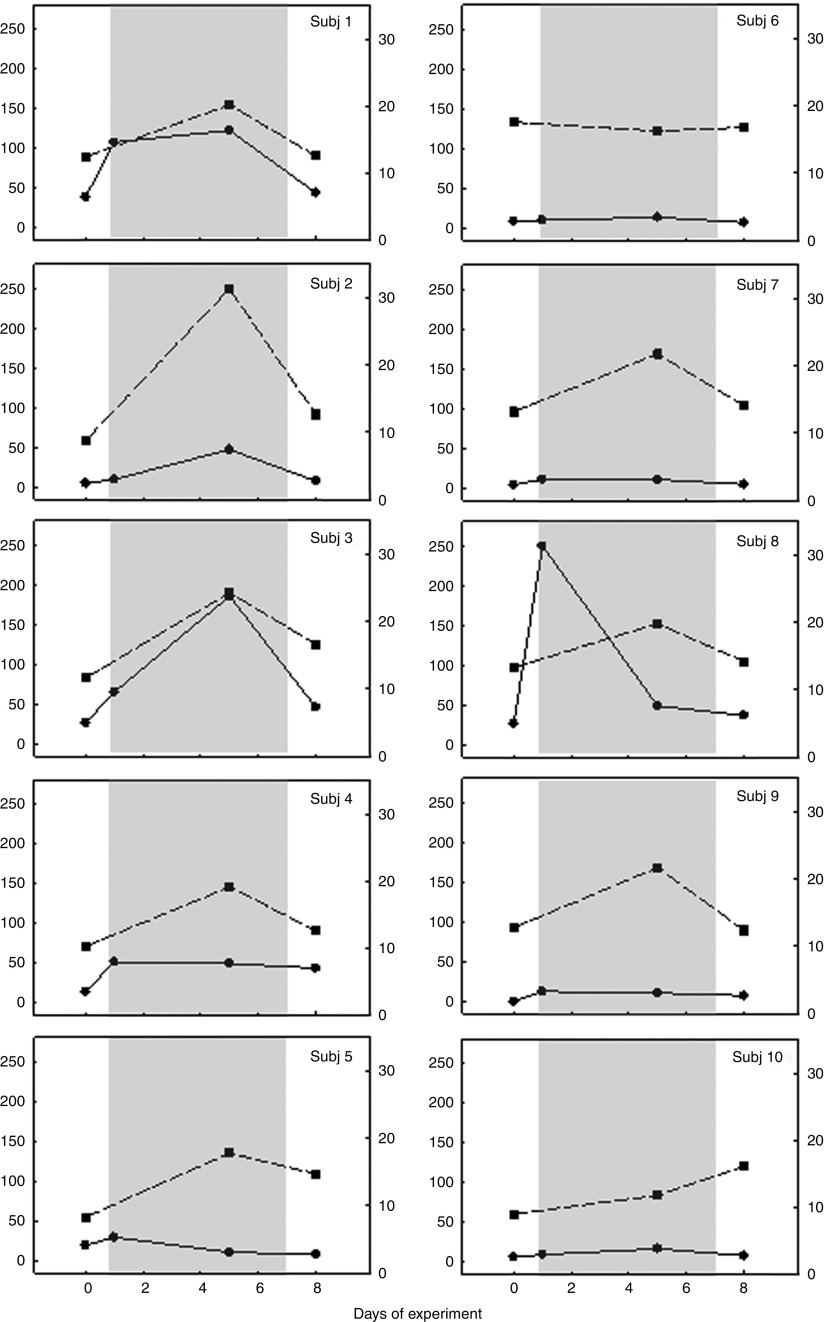
Inositol concentration in urine and blood of research staff volunteers. Inositol was quantified by MS^(^ ^18^ ^)^ in urine (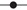, inositol concentration µm/mm creatinine) and venous blood plasma (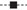, inositol concentration in blood (µm)) samples obtained from a group of ten volunteer subjects (Subj 1–10) who took daily inositol supplements (1·3 g) on days 1–7 of the experiment (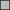). Urine was obtained on the day preceding the start of dosing (day 0), on days 1 and 5 of dosing (in each case 3–8 h after taking inositol) and on day 8 when 24 h had elapsed since the final dose. Blood samples were obtained at these same times on days 0, 5 and 8. Note the relatively uniform blood inositol concentration response of subjects to the dosing, whereas urinary excretion of inositol is highly variable between individuals. For example, Subj 1, 3 and 8 show marked (3–5-fold) increases in urinary inositol after dosing, whereas Subj 5, 6, 7, 9 and 10 show almost no increase in inositol excretion.

To further investigate the origin of individual variations in urinary inositol concentration, we obtained both urine and blood (plasma) samples from a group of young, adult research staff volunteers before, during and after a course of seven daily doses (1·3 g/d) of oral inositol supplementation. This study allowed us to measure blood concentrations compared with urinary inositol excretion in the same individuals over the same time period. It should be noted, however, that the volunteer study involved both men and women, unlike the pre-pregnancy study of women in the randomised trial group. Our findings (Fig. 3) revealed major variations between individuals in urinary inositol excretion: some subjects showed a more than 5-fold increase in urinary inositol concentration during the dosing period, with a return to pre-dosing levels 24 h after the final dose (Subj 1, 3, 8; Fig. 3), whereas others showed almost no elevation in urinary inositol concentration during dosing (Subj 5, 6, 7, 9, 10; Fig. 3). In contrast, these same subjects exhibited a much more uniform blood inositol concentration response to dosing, with nine of the ten individuals showing a 1·5–3-fold increase in inositol concentration during the dosing period, followed in most cases by a return to near pre-dosing levels 24 h after the final dose. Only one subject, who had the highest pre-dosing blood concentration, showed no increase during the dosing period (Subj 6; Fig. 3).

We conclude that, although urinary assay can detect usage of inositol supplements in some individuals, the marked inter-individual variation in urinary inositol clearance suggests that blood assay would be a more accurate method for future use.

## Discussion

### Prospects for a large-scale controlled trial of inositol in neural tube defects prevention

A goal of the PONTI pilot study was to evaluate the feasibility of recruiting women at high-risk of NTD to a controlled trial of inositol plus FA for prevention of NTD recurrence. On the basis of our UK data, a future, larger-scale clinical trial could expect to randomise fewer than half the eligible women who make contact. The reason for this is that many women are likely to opt for self-supplementation with inositol in addition to FA in their next pregnancy. This reflects many women’s over-riding desire to have a baby unaffected by NTD, which often appears to outweigh any concerns they might have about the lack of clinical trial evidence for the effectiveness of inositol in preventing NTD. The design and size of a future, larger-scale trial would therefore need to take this recruitment challenge into account.

A trial design involving randomisation of individual women to either inositol+FA or placebo+FA groups (as in the PONTI pilot study) would be the most statistically powered way to determine inositol’s efficacy in NTD prevention. Nevertheless, given the recruitment challenges we have identified, an alternative trial design might be preferable – for example, cluster randomisation could be considered in which all women in certain (randomly chosen) geographical locations would receive supplements containing inositol plus FA or FA alone during a specific trial period. Alternatively, a sequential study design could be considered, in which high-risk pregnancies are first studied under a regimen of FA only supplementation (i.e. the current clinical practice), after which all high-risk women planning a pregnancy would be prescribed inositol plus FA. In the case of FA for NTD prevention, although the most definitive evidence came from randomised controlled trials^(^ ^2^ ^,^ ^20^ ^)^, valuable supporting evidence also emerged from the China–U.S. Collaborative Project for Neural Tube Defect Prevention^(^ ^21^ ^)^, which compared NTD frequencies before and after introduction of peri-conceptional FA supplements. Whatever the trial design, it is likely that a multi-national collaborative effort would be needed to achieve the required number of supplemented high-risk pregnancies in a timely manner.

### Safety of inositol supplementation during pregnancy

Previous reports of inositol usage in human pregnancies at high risk of NTD did not detect any adverse effects on either mothers or babies^(^ ^15^ ^)^. In particular, there was no evidence of abnormal uterine contractions, suggested as a possible adverse effect of inositol therapy^(^ ^22^ ^)^. *Myo-*inositol has also been tested in adults for the prevention of depression, panic disorder and obsessive compulsive disorder^(^ ^23^ ^–^ ^25^ ^)^ and in children for the treatment of autism^(^ ^26^ ^)^. No significant side-effects were reported in these studies, which used relatively high inositol doses – up to 18 g/d in adults and 200 mg/kg in children.

In the PONTI pilot study, no adverse effects of the supplementation protocol were reported, either in mothers or babies. No unexpected complications of pregnancy were encountered, and neither did we record any unusual frequency of expected complications of pregnancy. For example, miscarriage occurred in 13 % (5/38) of our randomised pregnancies, consistent with estimates of up to 20 % miscarriage in normal populations^(^ ^27^ ^)^. Ectopic pregnancy occurs in 2 % of pregnancies, and our finding of one case gives a frequency of 2·6 %, close to the population average expectation. The frequency of pregnancies ending in caesarean section was similar in inositol and placebo groups. We conclude, therefore, that inositol supplementation during the peri-conceptional period does not appear to be strongly associated with any adverse outcomes for the mother or the baby.

### Neural tube defects recurrence frequencies and power calculation for a larger-scale clinical trial

There was one NTD recurrence among thirty-three randomised, established pregnancies in the PONTI pilot study, giving an NTD recurrence risk of 3·0 %. If we combine the randomised and non-randomised groups, there were three NTD recurrences among fifty-seven pregnancies – a 5·3 % recurrence risk. Typically quoted UK recurrence risks are 3·1 % for women following a single affected pregnancy and 11·8 % after two previous affected pregnancies^(^ ^16^ ^)^. Other NTD recurrence risk estimates from the genetic counselling literature include 3·4^(^ ^28^ ^)^, 2–5^(^ ^29^ ^)^, 3 (with 8 % risk after two affected pregnancies)^(^ ^30^ ^)^ and 2 % (with 10 % risk after two affected pregnancies, where the population incidence is 1/1000)^(^ ^31^ ^)^. Therefore, NTD recurrence rates in the PONTI study broadly resemble the literature values, consistent with most of our women subjects having experienced a single previous NTD pregnancy but some, particularly in the non-randomised group, having had two NTD.

The question arises as to why NTD recurrence risks in the PONTI pilot study, conducted between 2009 and 2013, resemble values that in some cases were based on clinical experience in the ‘pre-folic acid era’. One possibility is that most women in the UK do not take FA supplements peri-conceptionally. FA usage is known to be highly variable, particular in comparison between ethnic groups^(^ ^32^ ^,^ ^33^ ^)^, with the highest uptake among British Caucasian women. However, in our study, the great majority of women reported taking 0·4 mg FA in their earlier, NTD-affected, pregnancies, arguing against a lack of FA supplementation as an explanation. Moreover, some preventive action of FA might be expected in our study group, as the MRC Vitamin Trial^(^ ^2^ ^)^ found a significant reduction in recurrence in women with a similar pregnancy history of NTD. On the other hand, the MRC trial was conducted in the 1980s when most pregnancies in women with no history of NTD would probably have not been supplemented with FA, as its use was not widespread at the time. Therefore, some of the women with a previous NTD pregnancy who entered the MRC trial may have been FA-responsive but not ‘protected’ previously. In contrast, the great majority of women who entered our study had experienced an NTD despite taking 0·4 mg FA in their earlier pregnancy. One could argue that this group of women may have been particularly enriched for FA non-responsiveness, and thus might not have been particularly protected by FA usage in their subsequent pregnancy. Several studies have reported NTD persisting despite voluntary FA supplementation in a FA-fortified population^(^ ^5^ ^,^ ^6^ ^)^, leading to the conclusion that a significant proportion of NTD may be FA non-responsive^(^ ^34^ ^,^ ^35^ ^)^. Therefore, although such women will continue to have 4–5 mg FA prescribed for their subsequent pregnancies, it seems likely that additional supplement protection, perhaps by inositol, will be needed to reduce their otherwise high NTD recurrence risk.

The PONTI pilot study was not powered to detect a significant difference in NTD recurrence risk between the randomised groups. Indeed, the recurrence frequencies of 0 % (zero NTD/fourteen established pregnancies) in the inositol+FA group and 5·3 % (one NTD/nineteen established pregnancies) in the placebo+FA group generated a sample size of 150 (*P*=0·05; power=0·8) or 200 (*P*=0·05; power=0·9) in each trial arm. We note, however, that all three NTD recurrences recorded in this study (one in the placebo+FA randomised group and two in the non-randomised FA-only group) occurred in women not taking inositol. Larger patient groups will be required to achieve the statistical power needed to determine the efficacy of inositol supplementation on NTD recurrence.

### Monitoring compliance with supplementation

Although many clinical trials are analysed based on the principle of ‘intention to treat’, it can be beneficial to monitor compliance with the study intervention, in this case inositol supplementation. In the PONTI pilot study, we evaluated inositol assays using urine samples collected before and 6 weeks into the supplementation period. We tested urine rather than blood samples, as this greatly simplified the management of the study. The women subjects collected and sent in their own urine samples, making involvement of local medical staff unnecessary. In contrast, if blood sampling had been required, we would have needed to recruit and administer nearly as many local doctors/nurses as women subjects, as the latter were widely distributed across the UK.

Assay of inositol in urine samples was perfectly feasible, but revealed large inter-subject variations in inositol concentration. These variations seem unlikely to have resulted from non-compliance with the study protocol, as we also observed significant inter-subject variations in urinary inositol excretion in a group of research staff volunteers. In contrast, we found much more uniform blood (plasma) inositol responses between volunteers, following inositol supplementation. It appears, therefore, that urinary inositol measurement is insufficiently reliable to be used as a test of compliance for individual women in a trial setting. We would recommend blood inositol assay, for example, using dried blood spots, in a future, larger-scale clinical trial of inositol.

### Conclusions

Women at high risk of NTD recurrence can be recruited to a randomised trial structure, although fewer than half are likely to accept randomisation. Inositol supplementation during pregnancy has proven to be safe for both mothers and babies in our randomised study group. No NTD were encountered among fourteen randomised and twenty-one non-randomised pregnancies supplemented with inositol. In contrast, one NTD recurred among nineteen randomised and two NTD recurred among three non-randomised pregnancies that were supplemented with FA but not inositol. This study provides an impetus to further evaluate inositol for primary prevention of NTD.
